# Proteasomal degradation of polycomb-group protein CBX6 confers MMP-2 expression essential for mesothelioma invasion

**DOI:** 10.1038/s41598-020-72448-y

**Published:** 2020-10-07

**Authors:** Katsuya Sakai, Takumi Nishiuchi, Shoichiro Tange, Yoshinori Suzuki, Seiji Yano, Minoru Terashima, Takeshi Suzuki, Kunio Matsumoto

**Affiliations:** 1 Division of Tumor Dynamics and Regulation, Cancer Research Institute, Kanazawa University, Kanazawa, 920-1192 Japan; 2 WPI-Nano Life Science Institute (WPI-NanoLSI), Kanazawa University, Kanazawa, 920-1192 Japan; 3 Division of Functional Genomics, Advanced Science Research Center, Kanazawa University, Kanazawa, 920-0934 Japan; 4 Department of Medical Genome Sciences, Research Institute for Frontier Medicine, Sapporo Medical University School of Medicine, Sapporo, 060-8556 Japan; 5 Division of Medical Oncology, Cancer Research Institute, Kanazawa University, Kanazawa, 920-0934 Japan; 6 Division of Functional Genomics, Cancer Research Institute, Kanazawa University, Kanazawa, 920-1192 Japan; 7 Molecular Therapeutic Target Research Unit, Institute for Frontier Science Initiative, Kanazawa University, Kanazawa, 920-1192 Japan; 8 Tumor Microenvironment Research Unit, Institute for Frontier Science Initiative, Kanazawa University, Kanazawa, 920-1192 Japan

**Keywords:** Mesothelioma, Epigenetics, Mesothelioma

## Abstract

The aggressive invasiveness of malignant mesothelioma limits cancer therapy, however, the molecular mechanisms underlying the invasiveness remain largely unknown. Here we found that the matrix metalloproteinase-2 (MMP-2) was required for the invasion of mesothelioma cells in the collagen matrix and the gene expression of MMP-2 was correlated with the invasive phenotype. The MMP-2 gene expression was regulated by DNA and histone methylation around the transcription start site, implicating the involvement of the polycomb repressive complex (PRC). Knockdown of PRC component chromobox 6 (CBX6) promoted MMP-2 expression and invasion of mesothelioma cells. Transcriptome analysis suggested that CBX6 regulates sets of genes involved in cancer cell migration and metastasis. In invasive but not non-invasive cells, CBX6 was constantly unstable owing to ubiquitination and protein degradation. In human tissues, CBX6 localized in the nuclei of normal mesothelium and benign mesothelioma, but the nuclear staining of CBX6 was lost in malignant mesothelioma. These results suggest involvement of proteasomal degradation of CBX6 in mesothelioma progression.

## Introduction

Malignant pleural mesothelioma is characterized by extensive local growth and invasion of intrathoracic organs^1–3^. This is one of the reasons complicating surgical removal, thereby leading to poor prognosis. Matrix metalloproteinases (MMPs) are extracellular matrix remodeling endopeptidases implicated in pathological processes such as oncogenesis^4–6^. Secreted MMPs are inactive form (pro-MMPs) and proteolytic processing covert them to active form^4–6^. Active MMP-2 level was significantly elevated in malignant mesothelioma compared to uninflamed pleura, and this was correlated to poor prognosis^7^. The crucial involvement of MMP-2, MMP-9, and MT1-MMP in mesothelioma invasion has been investigated^8,9^, but the molecular events leading to the acquisition of the invasive behavior remains unknown. Studies using several cancer cell lines have strongly suggested the involvement of aberrant epigenetic regulations of MMP-2 in cancer progression^10–14^.

Chromobox 6 (CBX6) is one of CBX protein family, including CBX2, 4, 6, 7, and 8, which are the components of the polycomb repressive complex 1 (PRC1)^15^ and characterized as a transcriptional repressors^16^. CBX proteins regulate the stemness and differentiation of embryonic stem cells^17,18^ and hematopoietic stem cells^19^, whereas CBX proteins were dysregulated in cancers and it acts both as oncogene and tumor suppressor gene depending on the cell type^16^. For instance, CBX2 provided anoikis escape and dissemination in ovarian cancer^20^, CBX4 was overexpressed and promoted metastasis in osteosarcoma^21^, CBX6 suppressed proliferation, migration, and invasion in breast cancer^22^, and CBX7 inhibited cell migration in glioblastma^23^. The genomic and epigenomic factors relevant to malignant mesothelioma have been reported^3^, however, little is known about the involvement of CBX proteins in mesothelioma.

In this study, we found that CBX6 in PRC1 is constantly unstable due to ubiquitination and degradation in invasive mesothelioma cells. Knockdown of CBX6 in mesothelioma cells altered sets of genes that potentially participate in migration and invasiveness, including MMP-2. CBX6 shows nuclear localization in normal mesothelial cells, but it is largely diminished in the malignant mesothelioma in human patients, suggesting the clinical relevance of our findings.

## Results

### MMP-2 expression and function in mesothelioma invasion

**Figure 1:**
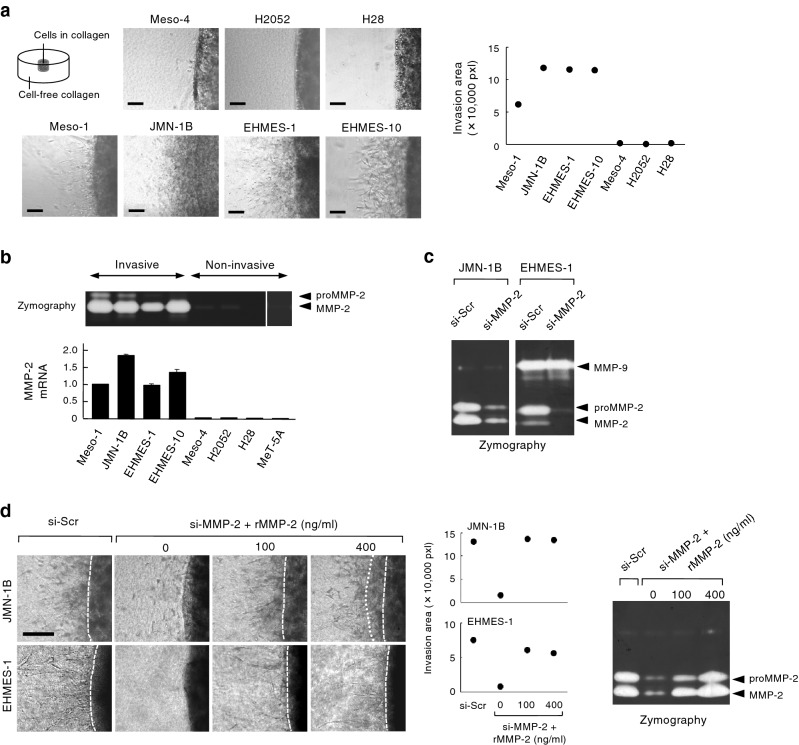
MMP-2 expression and function in mesothelioma invasion. (**a**) Mesothelioma cells in collagen matrix were non-invasive (Meso-4, H2052, H28) or invasive (Meso-1, JMN-1B, EHMES-1, EHMES-10). Scale bar: 200 μm. Invasion area was quantified by ImageJ. (**b**) MMP-2 activity and mRNA levels. MMP-2 activity in the cultured medium (48 h) of normal mesothelial cells (MeT-5A) and mesothelioma cells was detected by gelatin zymography. proMMP-2 indicates an inactive form. Relative mRNA levels normalized to GAPDH mRNA are represented as means + s.d. of three independent experiments. (**c**) MMP-2 knockdown in invasive cells treated with MMP-2 siRNA (si-MMP2) determined by zymography. (**d**) Inhibition and restoration of collagen invasion (left) and MMP-2 activity (right) in cells treated with si-Scr or si-MMP-2 and recombinant MMP-2 protein (rMMP-2). Invasion area was quantified by ImageJ. Scale bar: 200 μm.

We characterized seven human malignant mesothelioma cell lines for their invasive ability in type I collagen gel (Fig. 1a). The invasive cell lines were Meso-1, JMN-1B, EHMES-1 and EHMES-10 and the non-invasive cell lines were Meso-4, H2052, and H28. The invasion was blocked by GM6001, a selective inhibitor of MMPs (Fig. S1). Among MMPs capable of breaking type I collagen (MMP-2, MMP-8, MMP-13, and MT1-MMP), MMP-2 mRNA and protein were selectively detected in invasive but not in non-invasive mesothelioma cells (Fig. 1b, Fig. S2, MMP-13 mRNA was not detected). Small interference RNA (siRNA)-mediated knockdown of MT1-MMP responsible for activation of pro-MMP-2^6^ resulted in the failure of pro-MMP-2 activation in invasive cells (Fig. S3a). Invasive cells treated with siRNA targeting MT1-MMP lost their invasive ability (Fig. S3b). Furthermore, siRNA-mediated knockdown of MMP-2 in invasive cells (Fig. 1c) also resulted in the loss of invasive ability (Fig. 1d). Inversely, the addition of recombinant MMP-2 protein in the culture of si-MMP-2-treated cells recovered the invasive ability (Fig. 1d). Thus, MMP-2 is definitively responsible for collagen invasion of mesothelioma cells and MMP-2 expression is correlated with the invasive phenotype.

### CpG methylation of MMP-2 promoter is correlated with the invasive phenotype

**Figure 2:**
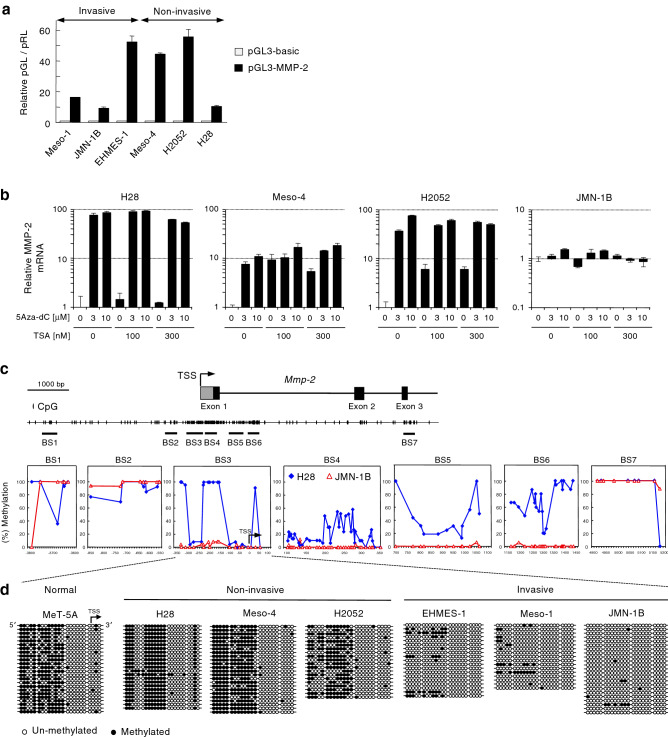
Epigenetic regulation of MMP-2 in mesothelioma cells. (**a**) Reporter gene analysis for the MMP-2 promoter. Relative reporter activity of pGL3-MMP-2 normalized to pGL3-basic is presented as means + s.d. of three independent experiments. (**b**) Changes in MMP-2 mRNA expression in non-invasive (H28, Meso-4, H2052) and invasive (JMN-1B) mesothelioma cells treated with 5-Aza-2′-deoxycytidine (5Aza-dC) and trichostatin A (TSA). The cells were treated with 5Aza-dC or vehicle for 5 days with drug refreshment every 24 h. The cells were treated with 5Aza-dC and TSA or vehicle for another 24 h. mRNA levels were quantified by qPCR. Relative mRNA levels normalized to GAPDH mRNA are represented as means + s.d. of three independent experiments. (**c**) Schematic representation of the transcription start site (TSS), CpG dinucleotides, and regions subjected to bisulfite sequencing (BS1 to BS6) in the MMP-2 gene. Methylation of CpG dinucleotides was determined by bisulfite sequencing in non-invasive (H28) and invasive (JMN-1B) cells. (**d**) Methylation of CpG dinucleotides at BS3 was determined by bisulfite sequencing in normal mesothelial cells (MeT-5A) and mesothelioma cell lines.

To determine mechanism for MMP-2 gene regulation, we first examined MMP-2 promoter activities by reporter gene assays in mesothelioma cells (Fig. 2a). Even in non-invasive mesothelioma cells wherein MMP-2 gene expression was silenced, MMP-2 promoter activities were considerably detected. These results suggest epigenetic silencing of MMP-2 promoter in non-invasive mesothelioma cells. To test this possibility, mesothelioma cells expressing MMP-2 (JMN-1B) or silenced for MMP-2 (H28, Meso-4, and H2052) were treated with an inhibitor of DNA methyl transferase, 5-Aza-2′-deoxycytidine (5Aza-dC), and an inhibitor of histone deacetylase, Trichostatin A (TSA), and then MMP-2 mRNA was quantified by PCR (Fig. 2b). 5Aza-dC-treatment alone significantly increased MMP-2 mRNA expression in H28, Meso-4, and H2052. TSA treatment, either in combination with 5Aza-dC or alone, increased MMP-2 mRNA expression in Meso-4 and H2052 cells. MMP-2 mRNA expression was not changed in JMN-1B cells treated with 5Aza-dC and/or TSA. Thus MMP-2 gene expression was epigenetically silenced in non-invasive mesothelioma cells.

We next analyzed the methylation status of CpG dinucleotides around the transcription start site (TSS) of MMP-2 gene in H28 (non-invasive) and JMN-1B (invasive) cells by bisulfite sequencing using seven sets of primers (BS1 to BS7 in Fig. 2c). In particular, the CpGs located approximately 130–230 nucleotides upstream of TSS in BS3 were highly methylated in H28 cells but not in JMN-1B cells. Importantly, CpGs in this region were hypermethylated in normal mesothelial cells (MeT-5A) and non-invasive mesothelioma cells (H28, H2052, Meso-4), while these were hypomethylated in invasive mesothelioma cells (JMN-1B, Meso-1, EHMES-1) (Fig. 2d), indicating a strong correlation with MMP-2 gene expression.

### Expression and CpG methylation of MMP-2 in patients with mesothelioma

**Figure 3:**
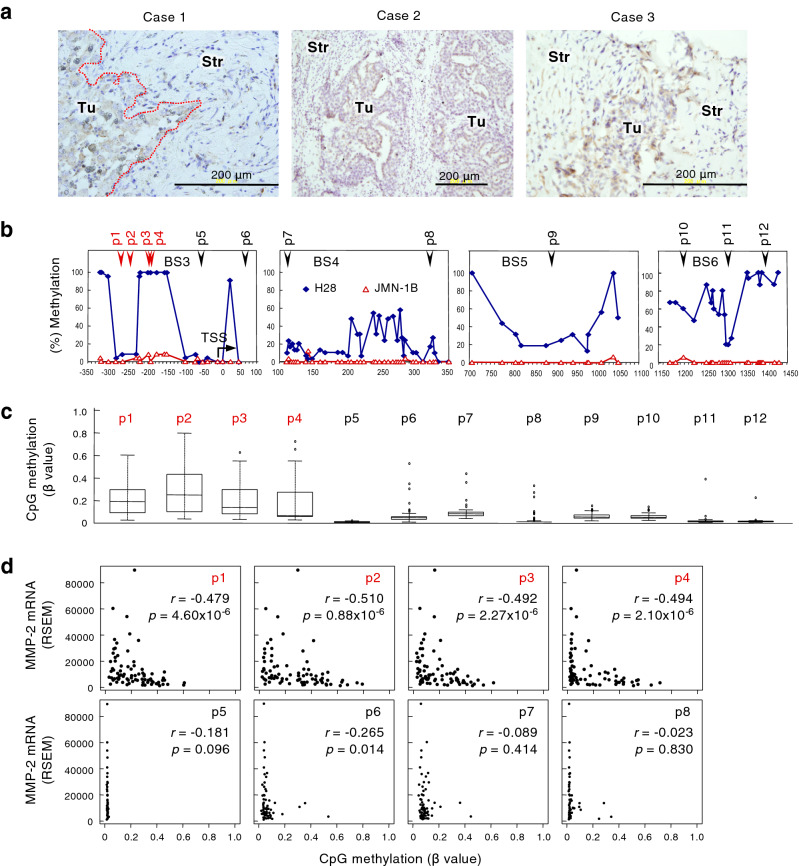
Expression and CpG methylation of MMP-2 in human clinical samples. (**a**) Immunohistochemical detection of MMP-2 in tumor tissues obtained from three patients with pleural malignant mesothelioma. Tu: tumor. Str: stroma. (**b**–**d**) CpG methylation status and their relationship to the expression levels of MMP-2 mRNA in tumor tissues from patients with malignant mesothelioma obtained from The Cancer Genome Atlas (TCGA) database. (**b**) Schematic representation of the positions of HM450 probes (p1 to p12) locating on MMP-2 locus. (**c**) The CpG methylation level at HM450 probes (p1 to p12). (**d**) Distributions of each relationship between CpG methylation status and expression levels of MMP-2. The *r* and *p* indicate Spearman’s correlation coefficient and associated P-value, respectively.

Immunohistochemical analysis of tumor tissues obtained from three patients with malignant mesothelioma indicated that MMP-2 was detected in mesothelioma cells rather than in stromal cells (Fig. 3a). We next analyzed The Cancer Genome Atlas (TCGA) database for CpG methylation at 12 probes localized between BS3 to BS6 (p1 to p12 in Fig. 3b) and MMP-2 mRNA expression levels in tissue samples obtained from 85 patients with malignant mesothelioma. CpG methylation status at p1 to p4 proximal to TSS—a region showing different methylation levels between invasive and non-invasive mesothelioma cells—indicated variation, while CpGs were hypomethylated in other regions (Fig. 3c). The scatter plot indicated that MMP-2 expression showed an inverse relationship to methylation status with statistical significance in p1 to p4 (Fig. 3d, upper panels). MMP-2 expression varied with no correlation to methylation status in p5 to p8 (Fig. 3d, lower panels). These results suggest the clinical relevance of MMP-2 expression and its regulation by CpG methylation proximal to transcription start site.

### CBX6 silenced MMP-2 in mesothelioma cells

**Figure 4:**
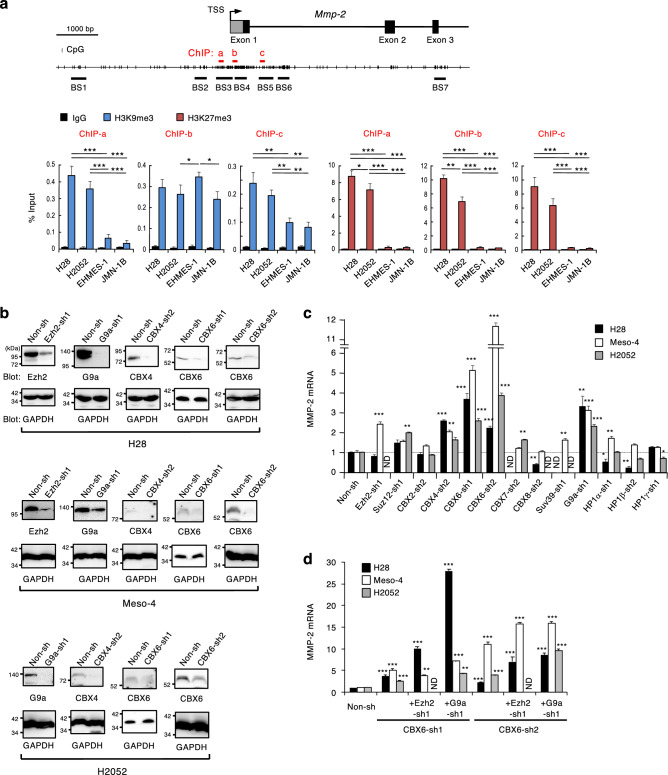
CBX6 silenced the MMP-2 gene in non-invasive mesothelioma. (**a**) Chromatin immunoprecipitation (ChIP) analysis for histone H3 around TSS of MMP-2 gene. The means + s.d. of four independent experiments are shown. ***p < 0.0001, **p < 0.01, and *p < 0.05. (**b**) Knockdown of target genes by stable expression of shRNAs targeting PcG was analyzed by western blot. Non-invasive mesothelioma cell lines (H28, Meso-4, H2052) were lentivirally transduced with shRNAs targeting indicated genes or control shRNA (Non-sh). Full membrane images are shown in Fig. S14. (**c**) Increase in MMP-2 mRNA expression levels by stable expression of shRNAs targeting PcG and related genes in non-invasive cells. MMP-2 mRNA was quantified by qPCR and normalized by β-actin mRNA. The means + s.d. of three independent experiments are shown. ***p < 0.0001, **p < 0.01 and *p < 0.05. ND: not determined. (**d**) Increase in MMP-2 mRNA expression levels by combined shRNA-mediated knockdown of CBX6 and EZH2 or G9a. The means + s.d. of three independent experiments are shown. ***p < 0.001 and **p < 0.01.

To further characterize histone modifications at MMP-2 promoter, we performed the chromatin immunoprecipitation (ChIP) assay for Histone H3 tri-methylated Lys9 (H3K9me3) and H3K27me3 (Fig. 4a). H3K27me3 was detected at higher level in non-invasive cells than in invasive cells at three tested ChIP regions. Since the repressive histone modification H3K27me3 is influenced by the function of PRC^15,16^, this result suggests the loss of PRC-mediated gene silencing in invasive mesothelioma. To specify molecules involved in MMP-2 gene silencing in polycomb group proteins (PcG), we examined the restoration of MMP-2 gene expression in non-invasive cells by lentiviral-mediated stable expression of short hairpin RNAs (shRNAs) targeting PcG. Knockdown of PRC2 components (EZH2 catalyzing H3K27 methylation; Suz12), PRC1 components (CBX2, CBX4, CBX 6, CBX 7, and CBX8 recognizing H3K27me3), and other related proteins (Suv39 and G9a catalyzing H3K9 methylation; HP1-α, HP1-β, and HP1-γ recognizing H3K9me3) were confirmed by qPCR or western blot (Fig. S4, S5 and Fig. 4b). The mRNA expression of MMP-2 was increased in these non-invasive cells (H28, Meso-4, H2052) by the knockdown of EZH2, CBX4, CBX6, and G9a alone (Fig. 4c). The combined knockdown of CBX6 with EZH2 or G9a further increased MMP-2 mRNA levels compared to the levels caused by the individual knockdown of EZH2, CBX6, and G9a (Fig. 4d). These results suggest that CBX6 suppressed MMP-2 expression in non-invasive cells, although the possible involvement of other CBX family is not excluded, because the knockdown efficiencies at RNA levels of CBX2, CBX7, CBX8 are not as good as those of CBX4 or CBX6 (Fig. S5).

To evaluate the target genes of CBXs, transcriptome of non-invasive mesothelioma (Meso-4) treated with shRNA targeting CBX6, CBX7, and CBX8 was analyzed by microarray. The gene expression profiles were in mostly non-overlapping manner, suggesting the difference of target genes (Fig. S6). Knockdown of CBX6 increased the expression levels of invasiveness-related genes such as *MMP-2*, adhesion molecules *ITGA1* and *SELL*, chemokine *CXCL2* (Fig. S6).

### Knockdown of CBX6 promoted MMP-2 expression and invasion of H2052 cells

**Figure 5:**
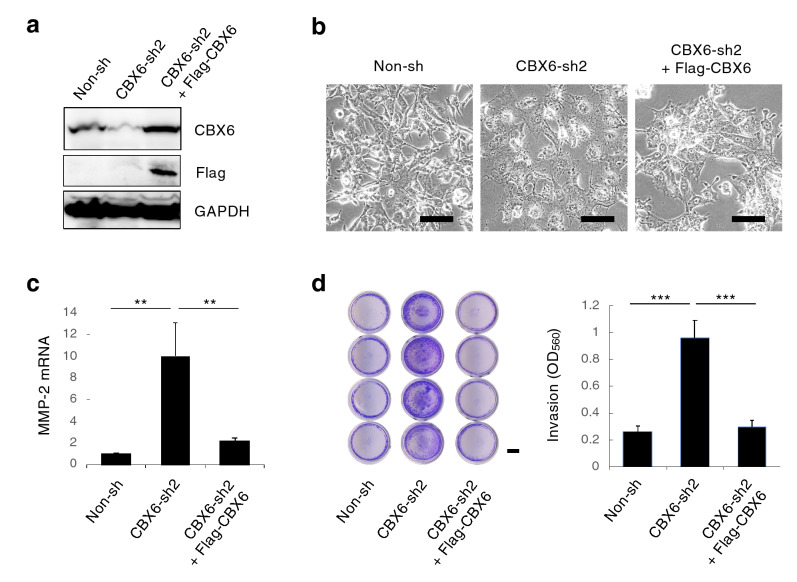
CBX6 suppressed invasion of mesothelioma. (**a**) Knockdown and restoration of CBX6 in H2052 mesothelioma cells analyzed by western blot. Non-invasive H2052 cells were lentivirally transduced with CBX6-target shRNAs (CBX6-sh2) or control shRNA (Non-sh). The established stable CBX6-knockdown H2052 cells were transfected with pcDNA3.1-Flag-CBX6 neo vector and selected by geneticin, resulted in the restoration of CBX6 expression (CBX6-sh2 + Flag-CBX6). Full membrane images are shown in Fig. S14. (**b**) The appearance of control H2052 (Non-sh), CBX6-knockdown (CBX6-sh2), and CBX6-restored (CBX6-sh2 + Flag-CBX6) cells. Scale bar: 100 μm. (**c**) MMP-2 mRNA levels in control H2052 (Non-sh), CBX6-knockdown (CBX6-sh2), and CBX6-restored (CBX6-sh2 + Flag-CBX6) cells. The means + s.d. (*n* = 5 for Non-sh and CBX6-sh2, *n* = 3 for CBX6-sh2 + Flag-CBX6, independent experiments). **p < 0.01. Unpaired two-tailed t-test. (**d**) Invasion of control H2052 (Non-sh), CBX6-knockdown (CBX6-sh2), and CBX6-restored (CBX6-sh2 + Flag-CBX6) cells. Scale bar: 2 mm. The means + s.d. (*n* = 4, distinct replicates for cell cultures). *** *p* < 0.001.

We next established H2052 cells stably knockdown the CBX6 by shRNA targeting CBX6, and recovered CBX6 expression by overexpression of Flag-CBX6 (Fig. 5a). The knockdown of CBX6 in H2052 cells changed the cells into flat and adhesive morphology (Fig. 5b), upregulated MMP-2 mRNA expression (Fig. 5c), and significantly increased the invasion (Fig. 5d). The overexpression of Flag-CBX6 in the CBX6-knockdown H2052 cells restored the morphology of H2052 cells (Fig. 5b), suppressed MMP-2 mRNA expression (Fig. 5c), and significantly reduced the invasion (Fig. 5d). Thus, the knockdown of CBX6 upregulated MMP-2 expression and mesothelioma invasion.

**Figure 6:**
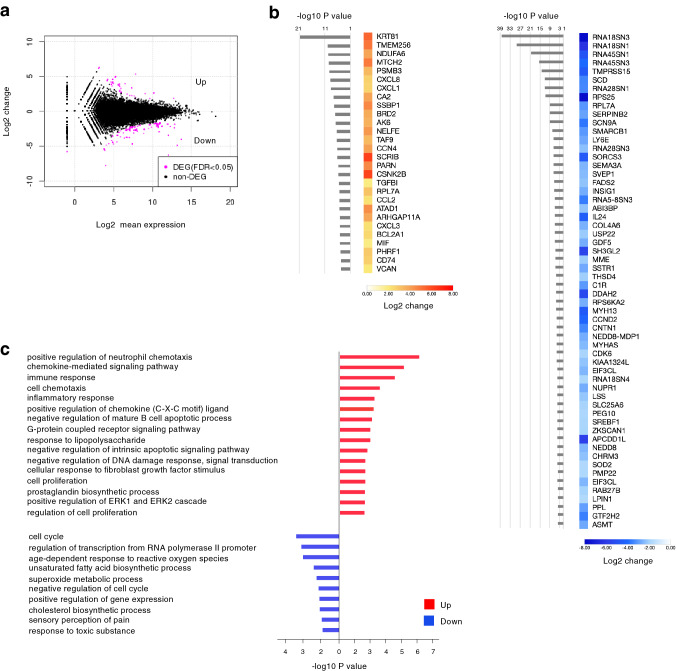
Effect of CBX6 knockdown on gene expression of H2052 cells. (**a**) MA plot representation of the RNA sequencing data, indicating differential expressed genes (DEG) between CBX6-knockdown and control H2052 cells. FDR: False discovery rate. (**b**) Heat map indicating the genes significantly up regulated (left) or down regulated (right) in CBX6-knockdown cells. (**c**) Gene ontology (GO) analysis for the significantly altered genes in CBX6-knockdown cells.

To identify potential target genes of CBX6, transcriptome of H2052 cells treated with control shRNA or CBX6-targeting shRNA was analyzed by RNA-seq (Fig. 6). We found 28 upregulated genes and 58 downregulated genes in the CBX6 knockdown condition (Fig. 6a,b). Unbiased functional enrichment analysis of the significantly differentially expressed genes in CBX6 knockdown condition showed an enrichment of various gene ontology (GO) terms including chemotaxis, immune response, negative regulation of apoptotic pathway, cell proliferation among the upregulated genes, while GO termes including cell cycle, various metabolic processes, and gene expression among the downregulated genes (Fig. 6c). Furthermore, we found upregulation of genes such as chemokines^24^ (*CXCL8*, *CXCL1*, *CCL2*, *CXCL3*), *SCRIB*^25^, *ARHGAP11A*^26^, *CCN4*^27,28^, *MIF*^29^, and *NELFE*^30,31^, which positively regulate migration and metastasis of various cancer cells, and also found downregulation of genes such as *SERPINB2*^32^, *ABI3BP*^33^, *COL4A6*^34^, *SH3GL2*^35^ and *ZKSCAN1*^36^,which negatively regulate migration and metastasis of various cancer cells.

### Degradation of CBX6 in invasive mesothelioma

**Figure 7:**
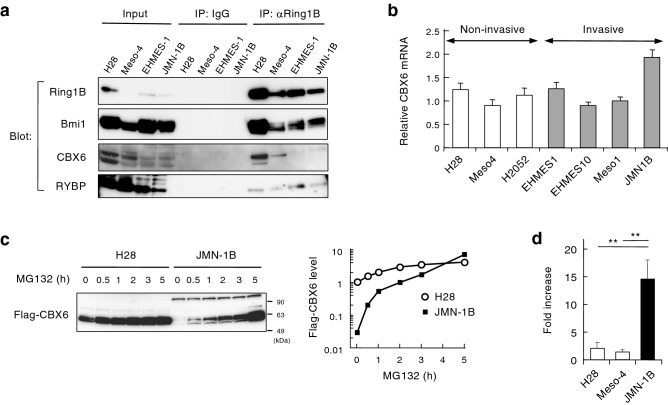
Degradation of CBX6 in invasive mesothelioma. (**a**) Association of CBX6 with PRC1 complex. Nuclear extracts prepared from non-invasive (H28, Meso-4) and invasive (EHMES-1, JMN-1B) mesothelioma cells were immunoprecipitated using anti-Ring1B antibody or control immunoglobulin G antibody (IgG). Nuclear extracts and immunoprecipitants (IP) were analyzed by western blot by using antibodies against CBX6, as well as antibodies against the core components of PRC1 (Ring1B, Bmi1, and RYBP). (**b**) Expression of CBX6 mRNA in mosothelioma cells. mRNA was quantified by qPCR and normalized by β-actin mRNA. The means + s.d. of three independent experiments are shown. (**c**) Enhanced degradation of CBX6 in invasive mesothelioma cells. Flag-tagged CBX6 was expressed in non-invasive (H28) or invasive (JMN-1B) mesothelioma cells. Cells were treated with 10 µM MG132 or left untreated. Whole cell lysates were subjected to western blot for anti-Flag antibody (left). Change in Flag-CBX6 protein levels determined by image analysis of western blot (right). (**d**) Fold increase in Flag-CBX6 protein level by MG132 treatment (5 h) in non-invasive (H28, Meso-4) and invasive (JMN-1B) cells. The means + s.d. of three independent experiments are shown. **p < 0.01. Full membrane images of Fig. 5a,c are shown in Fig. S15.

We then focused on the mechanism on how CBX6-mediated silencing might be impaired in invasive cells. Immunoprecipitation of endogenous Ring1B and detection of CBX6 and other core members of PRC1 (Bmi1 and RYBP)^15,16^ demonstrated the lack of CBX6 in PRC1 in invasive EHMES-1 and JMN-1B cells (Fig. 7a). Since CBX6 mRNA expression was comparable between non-invasive and invasive cells (Fig. 7b), we tracked the fate of Flag-tagged CBX6 stably expressed in non-invasive and invasive cells. Flag-CBX6 was mostly undetectable in invasive JMN-1B cells, whereas prevention of proteasomal degradation by MG132 treatment for 5 h restored the Flag-CBX6 by 14.6-fold (Fig. 7c,d). In contrast, Flag-CBX6 was detected even in the absence of MG132 in non-invasive H28 cells (Fig. 7c), and Flag-CBX6 level marginally increased by the treatment of MG132 for 5 h in non-invasive cells (Fig. 7d, 2.0-fold in H28. 1.3-fold in Meso-4). These results demonstrated that CBX6 degradation was enhanced in invasive cells.

### Domain mapping for degradation and ubiquitination of CBX6

**Figure 8:**
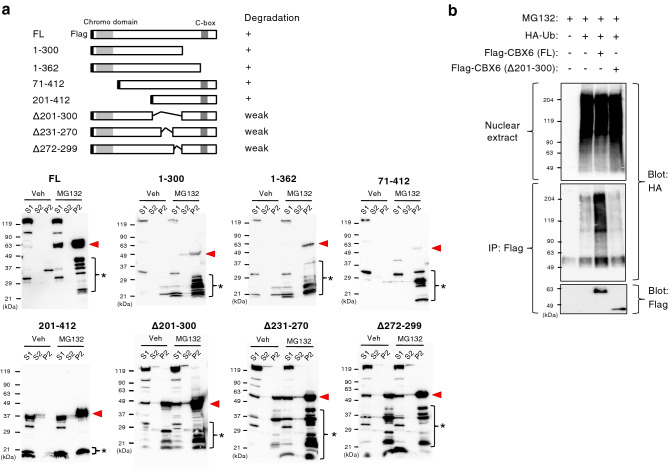
Domain mapping for degradation and ubiquitination of CBX6. (**a**) Schematic representation of CBX6 variants and summary of degradation in JMN-1B cells. FL: full length. Flag-tagged full length or truncation mutants of CBX6 were expressed in JMN-1B cells. Cells were treated with 10 µM MG132 or vehicle control (Veh) for 5 h and subjected to subcellular fractionation and western blot with anti-Flag antibody. S1: cytosol. S2: soluble nuclear. P2: chromatin. Red arrowheads indicate intact Flag-tagged CBX6. Asterisks indicate degraded fragment of Flag-tagged CBX6. (**b**) Ubiquitination of CBX6. HA-tagged ubiquitin (HA-Ub) and Flag-tagged full-length (FL) or deleted type (Δ201-300) CBX6 were expressed in JMN-1B cells. Cells were treated with 10 µM MG132 for 5 h; nuclear extracts were subjected to western blot or immunoprecipitation (IP, with anti-Flag antibody) and western blot with anti-HA or anti-Flag antibody. Full membrane images of Fig. 6b are shown in Fig. S15.

N-terminal chromodomains of CBX proteins interact with H3K27me3^37^. C-terminal polycomb repressor box (C-box) of CBX proteins interact with PRC1 components such as Ring1B^38^. To clarify the domains required for CBX6 degradation, we expressed the deletion forms of CBX6 in invasive JMN-1B cells and examined their stability and subcellular localization (Fig. 8a). Although full-length (FL) CBX6 was scarcely detected in vehicle-treated cells, it was clearly and predominantly detected in the chromatin fraction (P2) of MG132-treated cells (Fig. 8a, FL, red arrowhead). Deletion of chromodomain or C-box domain did not restore CBX6 in the absence of MG132 (Fig. 8a, 71–412 and 1–362, respectively), indicating that these domains are not required for CBX6 degradation. Deletion of amino acids 201 to 300, 231 to 270, and 272 to 299 restored CBX6 even in the absence of MG132 (Fig. 8a, Δ201-300, Δ231-270, Δ272-299, respectively), suggesting that the degradation of CBX6 is dependent on amino acids 201–300 in CBX6. Next, we analyzed ubiquitination of full-length CBX6 and CBX6Δ201-300 in nuclear extract of invasive JMN-1B cells treated with MG132. The results demonstrated ubiquitination of full-length CBX6, whereas lack of ubiquitination of CBX6Δ201-300 (Fig. 8b). These results demonstrated that the degradation of CBX6 was through ubquitin-modification on amino acids 201–300 in CBX6.

### Loss of nuclear CBX6 in malignant mesothelioma tissues

**Figure 9:**
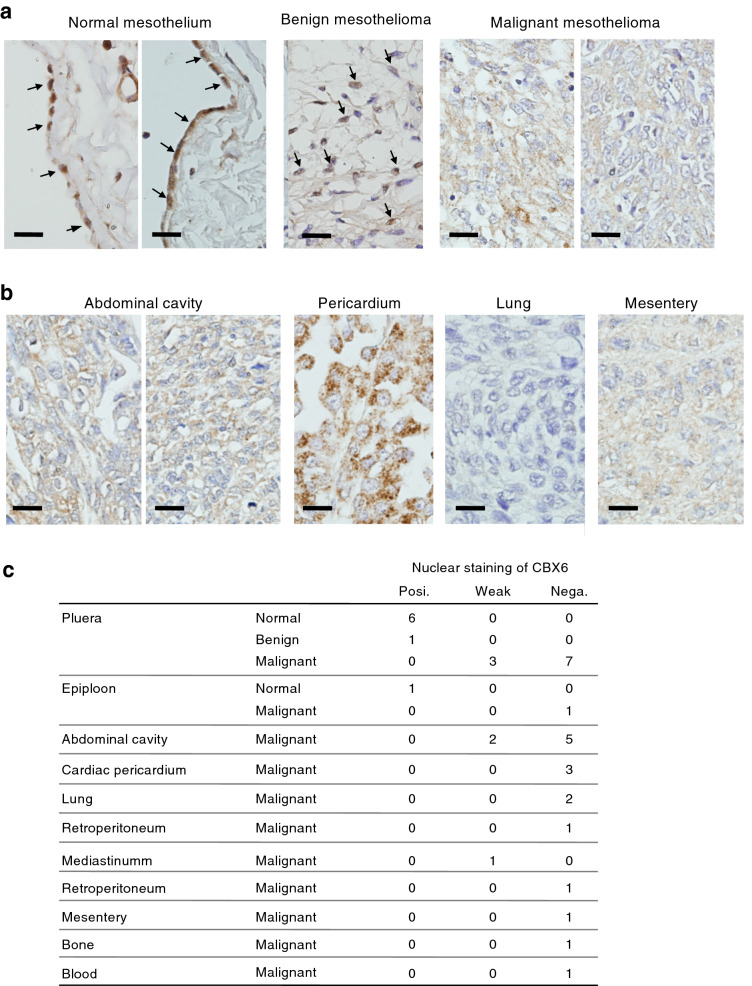
Immunohistochemical detection of CBX6 in human normal mesothelium and mesothelioma tissues. (**a**) Representative results of CBX6 immunohistochemistry in normal pleural mesothelium, benign pleural mesothelioma, and malignant pleural mesothelioma. Arrows indicate nuclear staining of CBX6. Scale bar: 20 μm. (**b**) Representative results of CBX6 immunohistochemistry in malignant mesothelioma of the abdominal cavity, pericardium, lung, and mesentery. Scale bar: 20 μm. Immunohistochemical results in other samples are shown in Fig. S7. (**c**) Summary of nuclear staining of CBX6. Samples (normal mesothelium, *n* = 7; benign mesothelioma, *n* = 1; malignant mesothelioma, *n* = 26) were classified into three groups, according to the nuclear staining for CBX6. Posi: strong nuclear staining. Weak: weak nuclear staining more than 50% cells. Nega: no or faint staining.

To test the clinical relevance of the loss of CBX6-mediated silencing in mesothelioma progression, we performed immunohistochemical staining of CBX6 in human normal mesothelium and mesothelioma tissues (Fig. 9*,* Fig. S7). Normal mesothelial cells in the pleura (6 cases) and the greater omentum (1 case) clearly showed nuclear staining of CBX6 (Fig. 9a,c*,* Fig. S7). Benign pleural mesothelioma also showed nuclear staining of CBX6 in the majority of the tumor cells (1 case, Fig. 9a). In contrast, nuclear staining of CBX6 was faint in 20 cases or weak in 6 cases in 26 cases of malignant mesothelioma of various tissue origins (Fig. 9a–c, Fig. S7). These results indicated that nuclear CBX6 of normal mesothelial cells was lost in the malignant progression of mesothelioma in human tissues.

## Discussion

Although MMP-2, MMP-9, and MT1-MMP have been implicated in mesothelioma invasion^8,9^, our results demonstrated that MT1-MMP-mediated MMP-2 activation is essential for collagen invasion and there is strong correlation between MMP-2 gene expression and invasive phenotype. The MMP-2 gene expression was regulated by CpG and histone methylation status around MMP-2 promoter. TCGA analysis of human mesothelioma samples further supported the correlation between CpG methylation status in this specified region and MMP-2 expression levels. Collectively, these results suggest that the dysregulation of epigenetic silencing of MMP-2 is involved in mesothelioma progression to invasive tumors.

Using shRNA-mediated knockdown of PcG proteins, we found CBX6 as a major PcG silenced MMP-2 in non-invasive mesothelioma cells. Enhanced degradation of CBX6 in invasive mesothelioma cells and loss of nuclear CBX6 during malignant progression of mesothelioma tissues suggests that the loss of CBX6 function may contribute to the malignant progression of mesothelioma. Since MMP-2 is essential for collagen invasion as we shown here, MMP-2 upregulation in invasive mesothelioma cells, which might be caused by CBX6 degradation, would contribute mesothelioma invasion. Transcriptome analysis showed significantly altered expression of sets of genes, which has been known to regulate migration and metastasis of various cancers, in CBX-6 knockdown condition. Although we have not tested functional involvement of these genes in mesothelioma invasion, they might contribute mesothelioma invasion. GO enrichment analysis showed upregulation of chemokines, which are involved in malignant progression of various cancers^24^, and positive regulation of cell proliferation, and negative regulation of apoptosis in CBX6-knockdown condition. While genes involved in cell cycle, transcription, and metabolic pathways were downregulated in CBX6-knockdown condition. In glioblastoma and breast cancer, CBX6 has been reported as a tumor suppressor^22,39^. CBX6 mRNA was downregulated as the astrocytoma grade increased and ectopic expression of CBX6 in glioblastoma cells decreased cell proliferation^39^. CBX6 mRNA was frequently downregulated in human breast cancer and ectopic expression of CBX6 reduced proliferation, migration, and invasion of breast cancer cells^22^. The relevance of CBX6 in other tumor types and other cellular contexts must be further studied.

In this study, we showed enhanced ubiquitination and degradation of CBX6 in invasive mesothelioma compared with that in non-invasive mesothelioma. De-ubiquitination of CBX4 and CBX6 by ubiquitin-specific protease 26 (USP26) stabilized these proteins and resulted in the suppression of pluripotency genes, demonstrating the regulation of the somatic reprogramming of embryonic stem cells by ubiquitin–proteasome degradation of the CBX proteins^40^. Recently, it was shown that T437 phosphorylation in CBX4 by casein kinase 1α (CK1α) facilitated CBX4 ubiquitination at both K178 and K280 and subsequent degradation of CBX4 in osteosarcoma^21^. Because CBX4 enhanced metastasis of osteosarcoma, CK1α-mediated degradation of CBX4 was proposed as a potential target to suppress the metastasis of osteosarcoma^21^. In contrast to CBX4, the mechanism of the degradation of CBX6 has not been elucidated. Here we showed the region of CBX6 required for ubiquitination and degradation is not conserved among other CBX families, suggesting that CBX6 has specific ubiquitin ligase(s). It is noteworthy that the phosphorylation at Ser301 and Ser303 of CBX6, which is adjacent to the region required for ubiquitination, has been detected in mass spectrometry analysis without functional validation^41^. Further studies on the post-translational regulation of CBX6 and other polycomb family proteins could reveal the cellular signals linked to epigenetic regulation. This could provide the therapeutic implications in the development of drugs, thereby achieving a selective control of epigenetic gene regulation.

## Methods

### Cell culture

The human mesothelial cell line MeT-5A was obtained from the American Type Culture Collection (ATCC). Human mesothelioma cell lines were obtained as follows. JMN-1B was obtained from the Brigham and Women’s Hospital Cell Culture Core Facility. H2052 and H28 were obtained from the ATCC. ACC-Meso-1 and ACC-Meso-4 were obtained from the RIKEN Cell Bank. EHMES-1 and EHMES-10 were kindly provided by Dr. Hamada (Ehime University, Japan). MeT-5A was cultured in medium 199 supplemented with 3.3 nM epidermal growth factor, 400 nM hydrocortisone, 870 nM insulin, and 10% fetal bovine serum (FBS). Other cell lines were cultured in Roswell Park Memorial Institute (RPMI) medium supplemented with 10% FBS.

### Reagents and antibodies

GM 6001 and structurally related negative control were obtained from Calbiochem. Aprotinin was obtained from Wako. Leupeptin and Pepstatin A were obtained from Peptide Institute Inc. 5Aza-dC was obtained from Sigma. TSA was obtained from Cayman Chemical. Recombinant human MMP-2 protein was obtained from R&D Systems. The following antibodies were obtained: anti-MT1-MMP antibody (AB815, Millipore); anti-glyceraldehyde-3-phosphate dehydrogenase (GAPDH) (14C10, Cell Signaling technology); anti-Ring1B (D22F2, Cell Signaling); anti-Bmi1 (D20B7, Cell Signaling technology); anti-CBX6 (E-16 or H-1, Santacruz, for western blot); anti-CBX6 (Bethyl Laboratories, for immunohistochemistry); anti-H3K9me3 (provided by Dr. Kimura^42^); anti-H3K27me3 (07-449, Millipore); anti-DDDDK (Flag)-tag (PM020, MBL); anti-hemagglutinin (HA)-tag (5B8, MBL); and anti-β-tubulin (T5168, Sigma).

### Invasion assay

The method used in this analysis has been described previously^43^. Briefly, mesothelioma cells were suspended at 1.5 × 10^5^ cells (for JMN-1B) or 1.2 × 10^5^ cells (for others) in 40 µL of 2.4 mg/mL neutralized type I collagen (BD Biosciences). The cell suspension in collagen was solidified at 37 °C for 90 min in a 96-well half area plate (Sigma) precoated with 3% bovine serum albumin. The solidified cell aggregates were embedded in 500 µL of 2.4 mg/mL neutralized cell-free type I collagen in a 24-well plate. The outer collagen was solidified at 37 °C for 60 min and then floated in 600 µL of RPMI1640 medium containing 1% FBS. The cells were cultured for 48 h, and the border between the inner cell aggregate and the outer gel were photographed. Alternatively, cell invasion was evaluated by CytoSelect 24-well Cell Invasion Assay (Cell Biolabs). H2052 cells were seeded at 1 × 10^5^ cells per insert in 200 μl RPMI1640 medium supplemented with 10% FBS, while 600 μl PMI1640 medium with 10% FBS was added to the bottom chamber. Cells were cultured for 72 h and fixed with 4% paraformaldehyde in PBS for 30 min. Cells invaded and attached to the bottom side of the membranes were stained with 0.4% crystal violet in 20% methanol and extracted with 100 μl Extraction Solution (Cell Biolabs). The optical density at 560 nm of the cell extractions were quantified by ARVO MX (Perkin Elmer).

### Quantitative PCR (qPCR)

Total RNA was extracted using the TRIZOL reagent (Invitrogen). First-strand cDNAs were synthesized using SuperScript III Reverse Transcriptase (Invitrogen) with a random hexamer. The primer sequences for mRNA quantification are listed in the Fig. S8. The PRISM 7,900 real-time PCR system (Applied Biosystems) and FastStart Universal SYBR Green Master (Roche) were used for the amplification and online detection. The amplified signals were confirmed to be single peak by dissociation curves and normalized to the levels of GAPDH or β-actin.

### Western blot and gelatin zymography

Cells were lysed in 20 mM Tris–HCl (pH 7.5), 1% (v/v) Triton X-100, 150 mM NaCl, 2 mM phenylmethylsulfonyl fluoride, 10 µg/mL aprotinin, pepstatin A, and leupeptin at 4 °C. Cell lysates were centrifuged at 10,000×*g* for 20 min at 4 °C and supernatants were collected. Protein concentration was determined using the DC protein assay reagent (BioRad), and equal amounts of proteins were subjected to sodium dodecyl sulfate–polyacrylamide gel electrophoresis (SDS-PAGE). The proteins were transferred onto polyvinylidene difluoride membranes using a semi-dry blotting apparatus (ATTO). Membranes were blocked and probed with the primary antibodies and horseradish peroxidase-conjugated secondary antibodies (Dako) (1:2000). Chemiluminescent signals were developed with Immunostar LD (WAKO) and observed using Image Reader LAS-3000 mini Ver. 2.2 (FUJIFILM). The same membranes were stripped and re-probed with the anti-GAPDH antibody. Conditioned media were collected from cell cultures in serum-free media and 20 µL of the samples was subjected to gelatin zymography, as described previously^6^.

### RNA interference

siRNA oligonucleotides were obtained from Nippon EGT. The siRNA sequences are listed in the Fig. S9. These siRNA duplexes (100 nM) were transfected into subconfluent cells by using Lipofectamine 2000 (Invitrogen). For shRNAs, the target sequences listed in Fig. S10 were cloned into the pLKO.1-puro vector (Sigma). Non-target shRNA vector (Sigma) was used as control. Lentiviruses were produced in 293 T cells transfected with packaging plasmids (Sigma-Aldrich) and shRNA constructs by using lipofectamine LTX (Invitrogen). Stable knockdowns in mesothelioma cells were selected with 1 µg/mL puromycin. Restoration of CBX6 was achieved by the overexpression of wild type Flag-CBX6. The established stable CBX6-knockdown H2052 cells were transfected with pcDNA3.1-Flag-CBX6 neo vector and selected by geneticin.

### MMP-2 promoter assay

MMP-2 genomic sequence, including promoter region^44,45^ (− 2049 to + 1, translation initiation site as + 1), was amplified by PCR using primers of 5′-ACGCGTGCTAGCCAAGGTTTGTCACTGGGTC-3′ (containing NheI site) and 5′-AATGCCAAGCTTATCGTAGCGCTCCCTGGC-3′ (containing HindIII site). The amplified human MMP-2 promoter region was subcloned into the NheI and HindIII site of pGL3-basic vector obtained from Promega (pGL3-MMP-2). Cells (1.5 × 10^6^) suspended in 400 µL of Opti-MEM (Invitrogen) were mixed with 1 µg of pRL-CMV vector (Promega) for internal control and 15 µg of pGL3-basic or pGL3-MMP-2 vector, and the vectors were introduced into the cells by electroporation at 220 V and 950 µF. The cells were cultured in a complete medium for 40 h and subjected to the Dual-Luciferase Reporter Assay System (Promega).

### Bisulfite sequencing

Cells were incubated for 48 h in 800 µL of lysis solution (50 mM Tris–HCl, 100 mM ethylenediaminetetraacetic acid, 0.5% SDS, 500 µg/mL proteinase K) at 55 °C. Genomic DNA was prepared with phenol/chloroform extraction, 2-propanol precipitation, and washing with 70% ethanol. Genomic DNA was subjected to sodium bisulfite modification using the methylSEQr Reagent Kit (Applied Biosystems). For analysis of DNA methylation, the human MMP-2 genomic regions were amplified by nested PCR. The primer sequences for nested PCR are listed in the Fig. S11. The PCR conditions used were as follows: initial denaturation at 94 °C for 5 min, 94 °C for 50 s, 51 °C for 2.5 min, and 65 °C for 2 min with a final extension at 65 °C for 10 min. The final PCR products were electrophoresed on 2% agarose gel. The expected bands were excised, purified using the QIAquick Gel Extraction kit (Qiagen), and TA cloned using the pGEM-T Easy Vector System (Promega) and sequenced.

### Human tissues and immunohistochemistry

Tumor specimens for MMP-2 immunostaining were obtained from three patients with mesothelioma, all of whom provided written informed consent at Kanazawa University in Japan. This study was approved by the Institutional Review Boards of Kanazawa University and all experiments were performed in accordance with the guidelines and regulations. Tissue arrays with normal mesothelium and benign and malignant mesothelioma for CBX6 immunostaining were obtained from Biomax. Paraffin-embedded 5-µm-thick sections were deparaffinized in xylene, rehydrated in decreasing concentrations of ethanol, and retrieved by autoclaving for 2 min at 105 °C with immersion in sodium citrate buffer (pH 6.0). After blocking the endogenous peroxidase activity with 3% H_2_O_2_ for 5 min, the sections were treated with 5% normal goat serum, 1% bovine serum albumin, and 0.2% Triton X-100 in phosphate-buffered saline (PBS). The sections were then reacted with anti-CBX6 rabbit polyclonal antibody (Bethyl Laboratories) at 1:1,000 dilution in 3% normal goat serum and 0.2% Triton X-100 in PBS at 4 °C overnight. After washing with PBS, the sections were treated with Histofine Simple Stain MAX PO (Nichirei Biosciences) for 60 min at room temperature. After washing with PBS, 3,3′-diaminobenzidine tetrahydrochloride (ImmPACT DAB, Dako) was used to detect immunostaining. Nuclei were counterstained with Mayer’s hematoxylin (WAKO).

### TCGA database

RNA sequencing data (Illumina HiSeq RNASeqV2 Level 3) containing 87 tumor samples and Infinium Human Methylation 450 K data (Level 3) containing 87 tumor specimens with clinical information were downloaded from TCGA database (https://cancergenome.nih.gov) in May 2016, respectively. Among them, 85 overlapping samples with mRNA expression data and DNA methylation data were used for further analysis. The IDs of the HM450 probes locating on MMP-2 locus used in this study are listed in Fig. S12. The correlations of MMP-2 expression and β-value of each probes are calculated with Spearman's Rank Correlation Coefficient. The β-value indicates the CpG methylation level as calculated by Intensity (methylated)/(Intensity(methylated) + Intensity(unmethylated)).

### ChIP

ChIP was performed as previously described^42,46^. Briefly, the cross-linked chromatins were immunoprecipitated with anti-H3K9me3 (provided by Dr. Kimura^42^), anti-H3K27me3 (Millipore), or normal mouse immunoglobulin G (IgG) (Jackson ImmunoResearch) bound to Dynabeads M-280 sheep anti-mouse IgG (Invitrogen). The enrichment of the specific amplified region by primers listed in Fig. S13 was analyzed by qPCR, and percentage enrichment of each histone modification over input chromatin DNA was shown.

### Microarray analysis

Microarray analysis were performed using the Whole Human Genome (8 × 60 k, Design ID 39494) Oligo Microarray according to the Agilent 60-mer Oligo Microarray Processing Protocol (Agilent Technologies). Total RNA samples (200 ng) were used to prepare Cy3-labeled cRNA using a Low RNA Input Fluorescent Linear Amplification Kit (Agilent Technologies). Fluorescence-labeled cRNAs were purified using an RNeasy RNA Purification Kit (Qiagen Inc., Hilden, Germany). Two independent RNA samples were used to confirm the reproducibility of the microarray analyses. The images were analyzed using the Feature Extraction Software (Ver. 10.7.3.1) and GeneSpring GX 12.1 software (Agilent Technologies). Normalization was performed as follows: (1) intensity-dependent Lowess normalization; (2) data transformation, with measurements set to ≤ 0.01; (3) per-chip 75th-percentile normalization of each array; and, (4) per-gene: normalized to the median of each gene. Genes differently expressed more than twice between Non-sh and shCBX6–8 treated Meso-4 cells were selected. The raw and processed data were deposited in the Gene Expression Omnibus database (access ID: GSE126605).

### RNA-Seq

RNA was prepared using the TRIZOL reagent (Invitrogen) and submitted to the Bioengineering Lab (Kanagawa, Japan), where cDNA libraries for RNA-Seq were prepared according to standard Illumina protocols. One hundred and fifty base pair-end read sequencing was performed on a DNBSEQ-G400 machine at the same facility. Reads were aligned to the reference human genome (GRCh38.p13 primary assembly) using the HISAT2 software. Differential expression gene (DEG) analysis was performed using iDEGES and edgeR. Results of differential analysis are provided in supplementary file S1. GO significantly altered in sample groups were identified by using DAVID.

### Immunoprecipitation of Ring1B

Anti-Ring1B antibody or rabbit IgG were chemically coupled on Magnosphere MS300/Carboxyl beads (JSR Corporation). Nuclear extracts were prepared using the NE-PER Nuclear and Cytoplasmic Extraction Reagents (Thermo Fisher Scientific). Immunoprecipitations were performed using 0.8 mg nuclear extracts with 10 µg antibodies in Nuclear Extraction Reagent (NER) supplemented with 20% glycerol, 0.5 mg/mL bovine serum albumin, 0.1% NP-40, 0.5 mM phenylmethylsufonyl fluoride, 1 µg/mL pepstatin A, 1 µg/mL leupeptin, and 1 µg/mL aprotinin. After incubation at 4 °C for 3 h, the beads were washed with NER 5 times and eluted with 50 mM Tris–HCl (pH 7.5), 1% SDS, and 300 mM NaCl. The eluates were mixed with SDS sample buffer and analyzed by SDS-PAGE, followed by western blot.

### CBX6 plasmids and ubiquitination assay

Full length and deletion mutants of CBX6 cDNA were prepared by the standard PCR procedure and cloned into the pLVSIN-CMV Pur vector (Takara) or pcDNA3.1(-) Neo vector (Thermo Fisher Scientific). The Flag-tag and 8 × glycine linker was appended to the N-terminus of CBX6 cDNA. All cDNAs were sequenced. pHA-Ub were obtained from Addgene. H28, Meso-4, or JMN-1B cells were transfected with pLVSIN-CBX6 plasmids using lipofectamine LTX and stably expressing cells were selected with 1 µg/mL puromycin. H2052 cells stably expressing shRNA targeting CBX6 (CBX6-sh2) were transfected with pcDNA3.1(-)-CBX6 plasmid using lipofectamine LTX and stably expressing cells were selected with 200 µg/mL neomycin. MG132 (10 µM) was added and cells were incubated for another 5 h. Nuclear extracts were prepared using the NE-PER Nuclear and Cytoplasmic Extraction Reagents and incubated with 5 µg anti-Flag-tag antibody (PM020, MBL) immobilized on Magnosphere MS300/Carboxyl beads. After incubation at 4 °C for 3 h, the beads were washed 5 times with NER and eluted with 1% SDS and 300 mM NaCl in 50 mM Tris–HCl (pH 7.5). The eluates were mixed with SDS sample buffer and analyzed by SDS-PAGE and western blot with antibodies against HA-tag (5B8, MBL) or Flag-tag.

### Statistical analysis

Two group comparisons were analyzed by a Student’s t-test using Prism 6 software. Values of *p* < 0.05 were considered statistically significant.

## Supplementary information

Supplementary Figure.

Supplementary Information.

## Data Availability

The authors declare that all data supporting the findings of this study are available within the article and its supplementary information files or from the authors upon reasonable request.
