# Supplementary material for: Proteasomal degradation of polycomb-group protein CBX6 confers MMP-2 expression essential for mesothelioma invasion
Source: Sci Rep. 2020 Oct 7;10:16678. doi: 10.1038/s41598-020-72448-y (PMC7541533; doi:10.1038/s41598-020-72448-y)
Supplement: Supplementary file 1 — Supplementary Figure. [file 41598_2020_72448_MOESM1_ESM.pdf]

Proteasomal degradation of polycomb-group protein CBX6 confers MMP-2 expression essential for mesothelioma invasion

Katsuya Sakai, Takumi Nishiuchi, Shoichiro Tange, Yoshinori Suzuki, Seiji Yano, Minoru Terashima, Takeshi Suzuki, and Kunio Matsumoto

Supplementary data

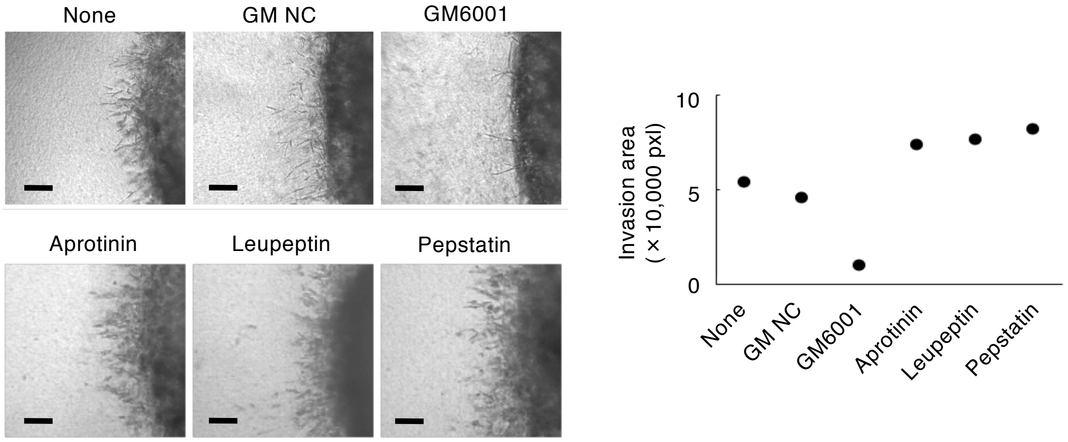

Fig. S1. Collagen invasion of EHMES-10 cells treated with protease inhibitors. GM NC: structurally related negative control of GM6001. GM6001: a broad inhibitor of MMPs. Scale bar: 200 µm. Invasion area was quantified by ImageJ.

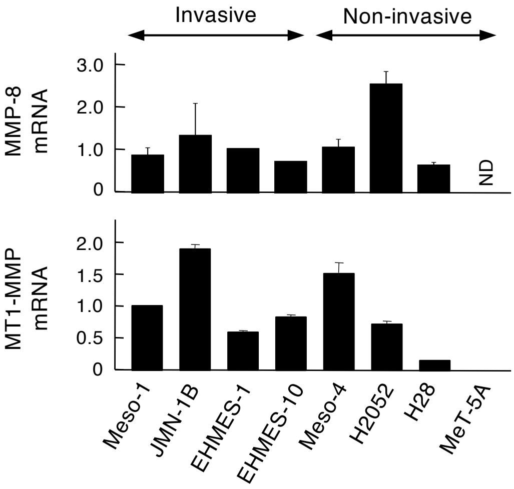

Fig. S2. Expression levels of MMP-8 and MT1-MMP mRNA. mRNA level was quantified by qPCR. Relative mRNA levels normalized to GAPDH mRNA are represented as means + s.d. of three independent experiments. ND: not determined.

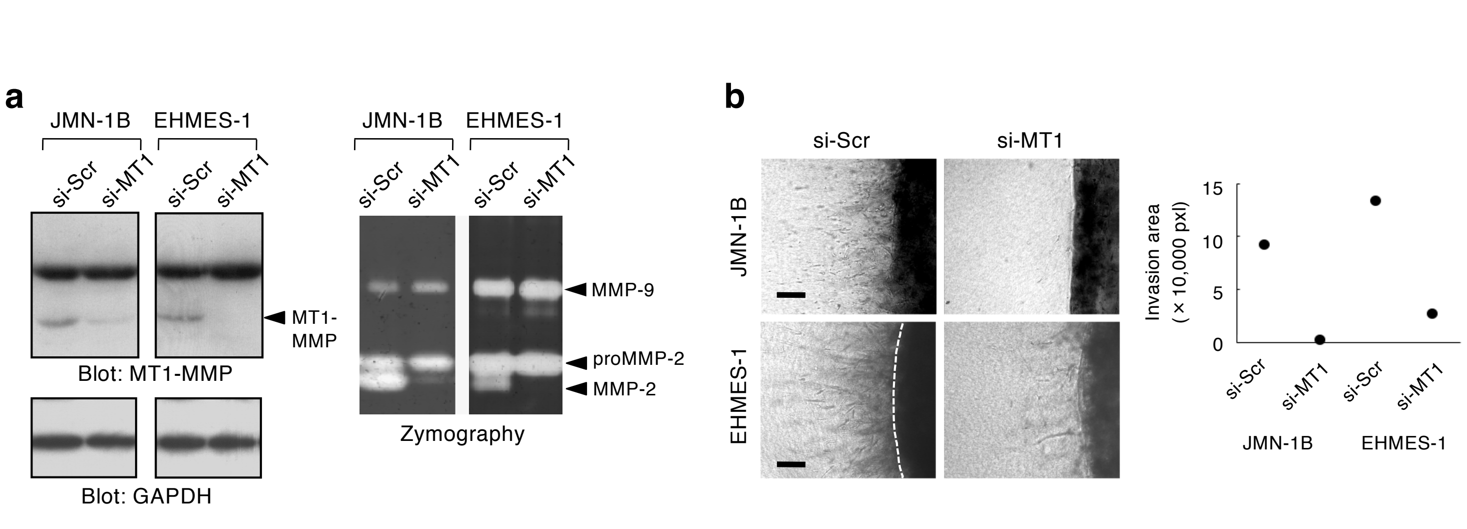

Fig. S3. MT1-MMP-dependent MMP-2 activation in invasive cells. (**a**) Cells were treated with control siRNA (si-Scr) or anti-MT1-MMP siRNA (si-MT1). MT1-MMP protein levels and MMP-2 activity were measured by western blot (left) and zymography (right, 24 h culture), respectively. (**b**) Collagen invasion of cells treated with si-Scr or si-MT1. Scale bar: 200 µm. Invasion area was quantified by ImageJ.

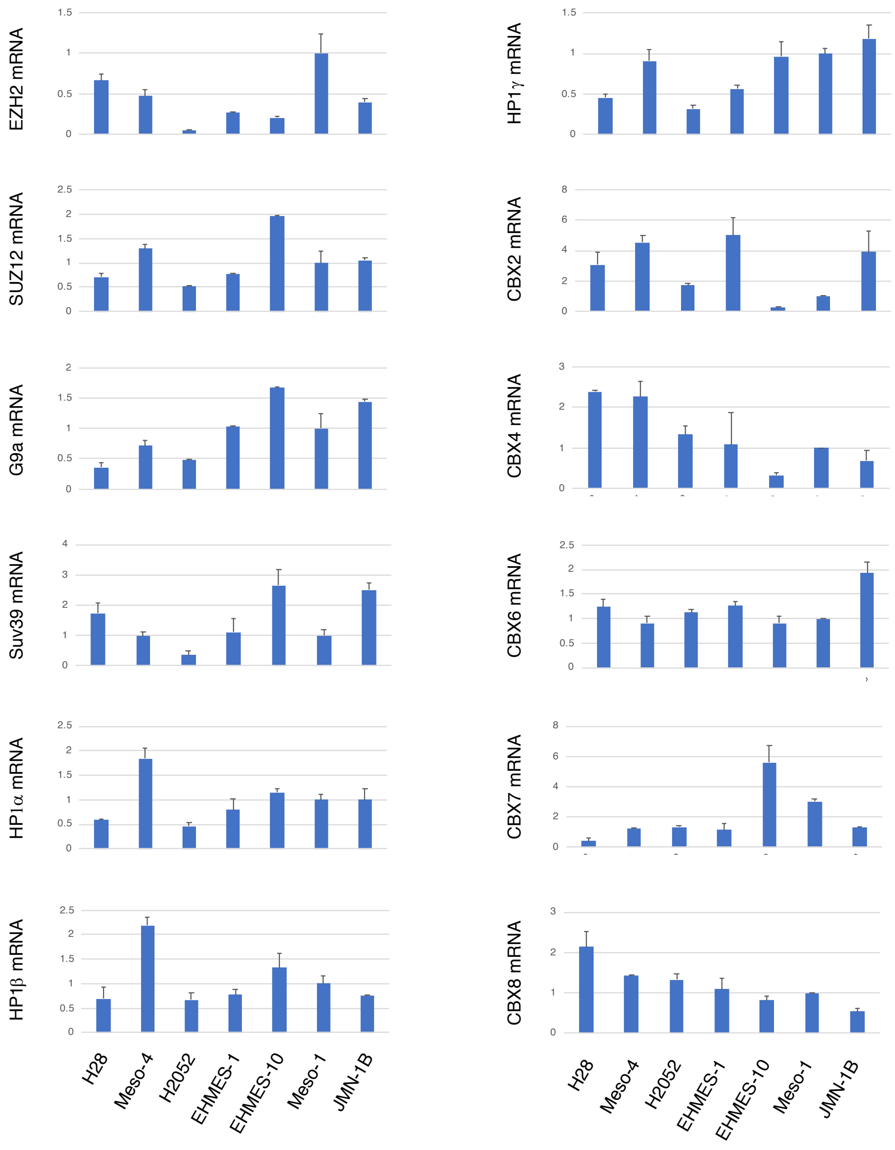

Fig. S4. mRNA expression of PcG genes and related genes in mesothelioma cells. mRNA was quantified by qPCR and normalized by β-actin mRNA. The means + s.d. of three independent experiments are shown.

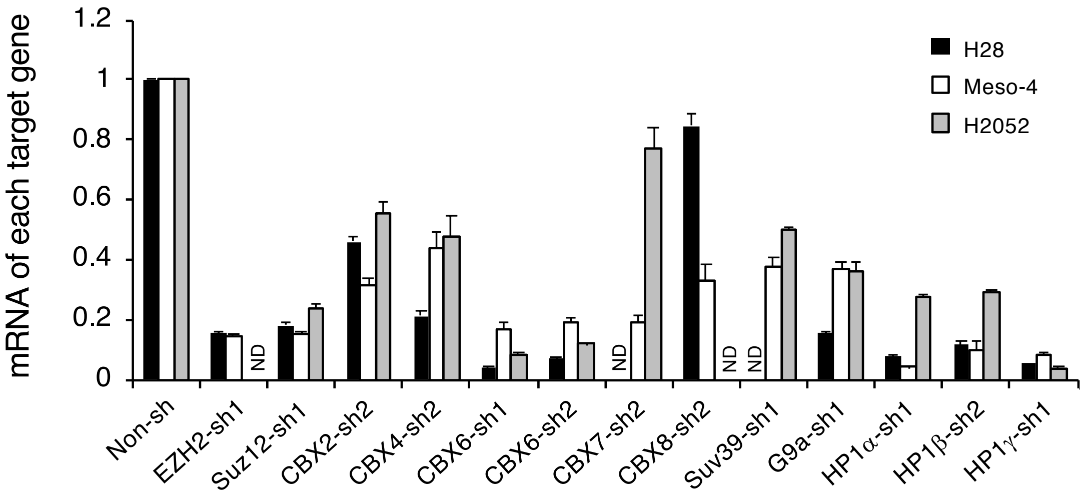

Fig. S5. Knockdown of PcG and related genes by shRNA was confirmed by qPCR. Non-invasive mesothelioma cell lines (H28, Meso-4, H2052) were lentivirally transduced with shRNA targeting each PcG gene and related gene indicated or non-target shRNA (Non-sh). mRNA of target genes were quantified by qPCR and normalized by β-actin mRNA. Relative amount of mRNA of each target gene were shown as means + s.d. of three independent experiments. ND: not determined.

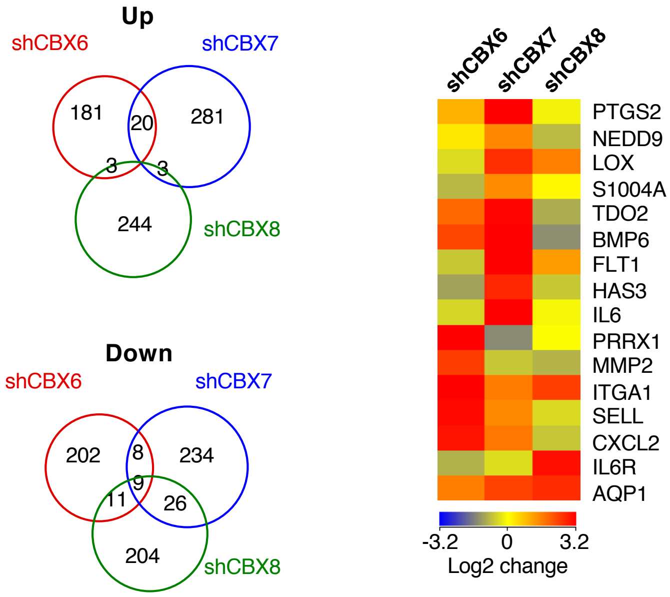

Fig.S6 Target gene selectivity of CBX proteins. Non-invasive Meso-4 cells stably expressing shRNAs for non-target or for CBX6, CBX7, or CBX8 were subjected to gene expression microarray analysis. Genes whose expression levels were changed by shRNA-mediated knockdown (fold change >2, $p < 0.05$) were selected and showed by Venn-diagram (left). Heat map of tumor-related genes that are upregulated by CBX6, CBX7, and CBX8 knockdown in Meso-4 cells (right, fold change >2, $p < 0.05$).

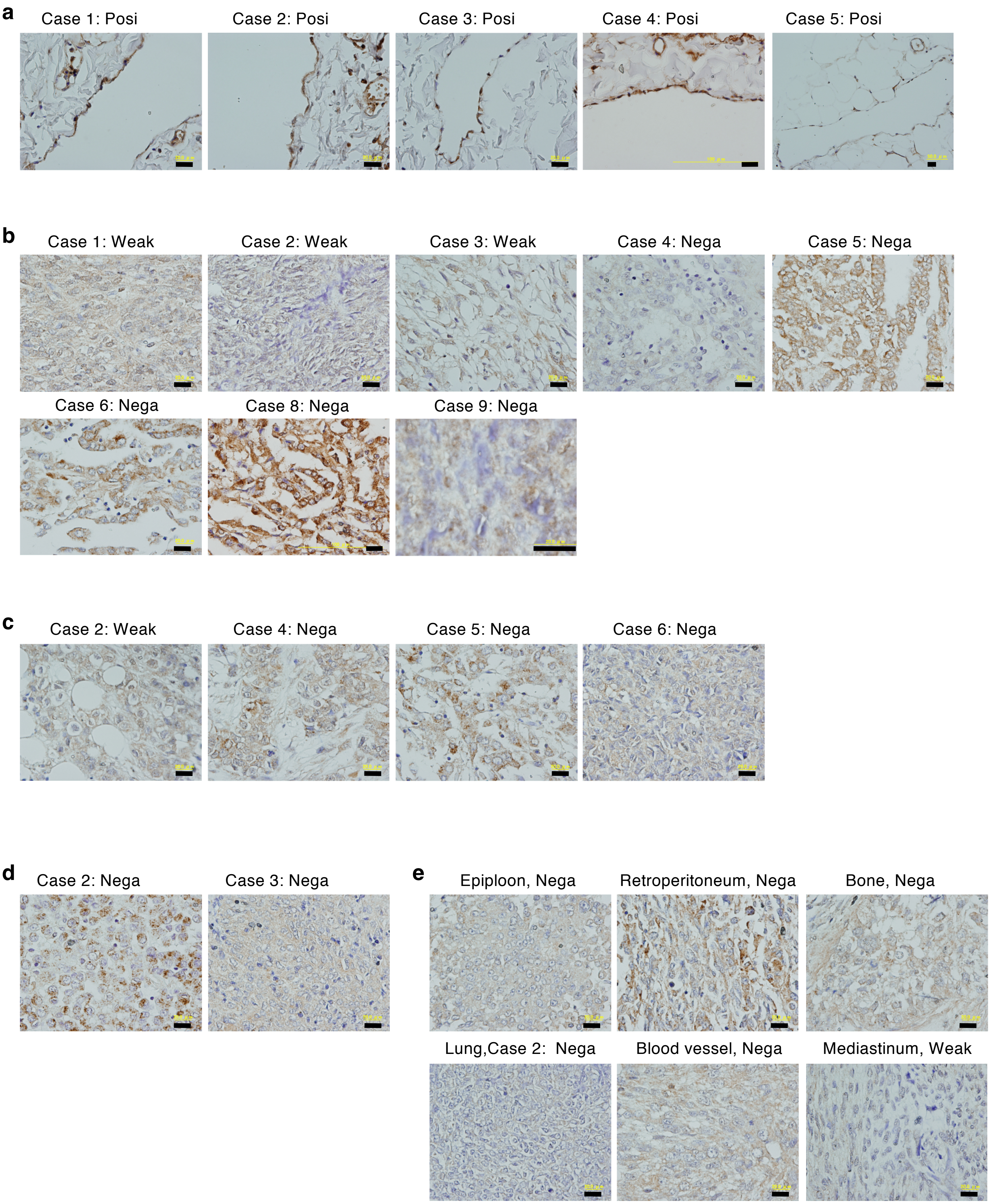

Fig. S7. Immunohistochemical detection of CBX6 in human normal mesothelium and mesothelioma tissues. (**a**) Normal pleural mesothelium. (**b**) Malignant pleulral mesothelioma. (**c**) Malignant abdominal mesothelioma. (**d**) Malignant cardiacpericardium mesothelioma. (**e**) Malignant mesotheliom in other tissues. Scale bar: 20 μm.

GAPDH
5′-GAGTCAACGGATTTGGTCGT-3′ (forward)
5′-GACAAGCTTCCCGTTCTCAG-3′ (reverse)

β-actin
5′-GGACTTCGAGCAAGAGATGG-3′ (forward)
5′-AGCACTGTGTTGGCGTACAG-3′ (reverse)

MT1-MMP
5′-CACTGCCTACGAGAGGAAGGCACTGC-3′ (forward)
5′-TTGGGGTACTCGCTATCCAC-3′ (reverse)

MMP-2
5′-ATGACAGCTGCACCACTGAG-3′ (forward)
5′-ATTTGTTGCCCAGGAAAGTG-3′ (reverse)

MMP-8
5′-TCTGCAAGGTTATCCCAAGG-3′ (forward)
5′-ACCTGGCTCCATGAATTGTC-3′ (reverse)

MMP-13
5′-TTGAGCTGGACTCATTGTCG-3′ (forward)
5′-GGAGCCTCTCAGTCATGGAG-3′ (reverse)

EZH2
5′-AGGAGTTTGCTGCTGCTCTC-3′ (forward)
5′-CCGAGAATTTGCTTCAGAGG-3′ (reverse)

Suz12
5′-CTTCCAGCCAGAAGAAAACG-3′ (forward)
5′-GTTTGGCAATAGGAGCCGTA-3′ (reverse)

CBX2
5′-GGCTGGTCCTCCAAACATAA-3′ (forward)
5′-ACATGGCAGTGAGCTTCCTT-3′ (reverse)

CBX4
5′-CCGAGGTCATCCTGCTAGAC-3′ (forward)
5′-CAAAGAAGGGCTTGAACTCG-3′ (reverse)

CBX6
5′-GAACCGCGTTATAGGCAAGA-3′ (forward)
5′-GGGTCAGAGGACTGTGGTGT-3′ (reverse)

CBX7
5′-GCTGGTTTTCCCCTTTTCTC-3′ (forward)
5′-AAGCCTCTCTGAAGGGGAAG-3′ (reverse)

CBX8
5′-AACTGGCTGTGGACACCTTC-3′ (forward)
5′-CCACCACCTTCTCCAGGTTA-3′ (reverse)

Suv39h1
5′-GCTATGACTGCCCAAATCGT-3′ (forward)
5′-ACGAAGCTGTTCTTGCGAAT-3′(reverse)

G9a
5′-CCTGATGGTGCTCTGTGAGA-3′ (forward)
5′-CATCCCATTCAGCTGAGACA-3′ (reverse

HP1α
5′-TGGAAAGGCTTTTCTGAGGA-3′ (forward)
5′-ATGTCATCGGCACTGTTTGA-3′ (reverse)

HP1β
5′-GTGGTAAAGGGCAAAGTGGA-3′ (forward)
5′-CCTCGTGGCTTTTCTGACTC-3′ (reverse)

HP1γ
5′-GCTGGCAAAGAAAAAGATGG-3′ (forward)
5′-TCAAGACCTCTGGCAAATCC-3′ (reverse)

Fig. S8. Primers for mRNA quantification by qPCR.

si-MT1-MMP (si-MT1)
5′-CAGGCAAAGCUGAUGCAGAtt-3′ (sense)
5′-UCUGCAUCAGCUUUGCCUGtt-3′ (antisense)

si-MMP-2
5′-GGAUGGCAAGUACGGCUUCtt-3′ (sense)
5′-GAAGCCGUACUUGCCAUCCtt-3′ (antisense)

Scrambled control (si-Cont)
5′-GUGAUCAAGCACCGAAGAGtt-3′ (sense)
5′-CUCUUCGGUGCUUGAUCACtt-3′ (antisense)

Fig. S9. siRNA oligos.

EZH2-sh1: 5′-CCGGAAACAGCTGCCTTAGCTTCACTCGAGTGAAGCTAAGGCAGCTGTTTCTTTTTG-3′

Suz12-sh1: 5′-CCGGGCTTACGTTTACTGGTTTCTTCTCGAGAAGAAACCAGTAAACGTAAGCTTTTTG-3′

CBX2-sh2: 5′-CCGGCGCCGAGTGCATCCTGAGCAACTCGAGTTGCTCAGGATGCACTCGGCGTTTTT-3′

CBX4-sh2: 5′-CCGGCGTGATCGTGATGAGCAAATACTCGAGTATTTGCTCATCACGATCACGTTTTTG-3′

CBX6-sh1: 5′-CCGGGCGTACACAGATCCGCCACATCTCGAGATGTGGCGGATCTGTGTACGCTTTTT-3′

CBX6-sh2: 5′-CCGGCCGCATCAGTGATGTGCATTTCTCGAGAAATGCACATCACTGATGCGGTTTTT-3′

CBX7-sh2: 5′-CCGGCGTGACCGACATCACCGCCAACTCGAGTTGGCGGTGATGTCGGTCACGTTTTT-3′

CBX7-sh3: 5′-CCGGCGTGACCGACATCACCGCCAACTCGAGTTGGCGGTGATGTCGGTCACGTTTTT-3′

CBX7-sh4: 5′-CCGGCCTCAAGTGAGGTGACCGTGACTCGAGTCACGGTCACCTCACTTGAGGTTTTT-3′

CBX8-sh2: 5′-CCGGACTTACGAGTTTCGAAGTGACCTCGAGGTCACTTCGAAACTCGTAAGTTTTTTG-3′

CBX8-sh3: 5′-CCGGACTTACGAGTTTCGAAGTGACCTCGAGGTCACTTCGAAACTCGTAAGTTTTTTG-3′

CBX8-sh4: 5′-CCGGGAAAGAGAGATGGAGCTCTATCTCGAGATAGAGCTCCATCTCTCTTTCTTTTT-3′

Suv39h1-sh1: 5′-CCGGGCAGGTGTACAACGTCTTCATCTCGAGATGAAGACGTTGTACACCTGCTTTTTTG-3′

G9a-sh1: 5′-CCGGCGAGAGAGTTCATGGCTCTTTCTCGAGAAAGAGCCATGAACTCTCTCGTTTTTG-3′

HP1α-sh1: 5′-CCGGGCCGATGACATCAAATCTAAACTCGAGTTTAGATTTGATGTCATCGGCTTTTTG-3′

HP1β-sh2: 5′-CCGGCCCACAGGTTGTCATATCCTTCTCGAGAAGGATATGACAACCTGTGGGTTTTTG-3′

HP1γ-sh1: 5′-CCGGGCGTTTCTTAACTCTCAGAAACTCGAGTTTCTGAGAGTTAAGAAACGCTTTTTG-3′

Fig. S10. shRNA sequences.

BS1
Outer: 5′-GGTAGTTATTAAAAGTAGGAG-3′ (forward), 5′-TAAACTTCTAATCTCAAAACTCT-3′ (reverse)
Nested: 5′-TTGGGAAGGTTTATATGGTA-3′ (forward), 5′-AACCACCCAATCTATAATACT-3′ (reverse)

BS2
Outer: 5′-ATTGTTATGGTATTGGTGG-3′ (forward), 5′-AATCCCAAATTACTTCCTTAC-3′ (reverse)
Nested: 5′-ATGAGTAGTGAGGATGATTA-3′ (forward), 5′-CCTCTTTCCTTCTACAAATA-3′ (reverse)

BS3
Outer: 5′-TTGTGGTTGATTATTTGTTTTTG-3′ (forward), 5′-TCCTAACAATCCCTTTATATATT-3′ (reverse)
Nested: 5′-GATTTTAGGGAGTGTAGGG-3′ (forward), 5′-CAACCTCCAACCACC-3′ (reverse)

BS4
Outer: 5′-TTGGAGGTTGY(C/T)GTATTTG-3′ (forward), 5′-ACCAACTCTTTATCCR(A/G)TTTTAA-3′ (reverse)
Nested: 5′-ATATAAAGGGATTGTTAGGATTTG-3′ (forward), 5′-TCAACAAACAACCCAAAA-3′ (reverse)

BS5
Outer: 5′-TGGTAAGTTATTGGAGTGAT-3′ (forward), 5′-CAATCACTTCCCAAATAAC-3′ (reverse)
Nested: 5′-GTAATTTTTAGTATATAGTAGTGG-3′ (forward), 5′-AACTATTTATTTAAAAATCCCTCTC-3′ (reverse)

BS6
Outer: 5′-GAAGTGATTGGGGTAG-3′ (forward), 5′-CCTAACCTACCTCTCT-3′ (reverse)
Nested: 5′-ATGGATAAATTGGTTAAGGG-3′ (forward), 5′-CCTCAAACCCTACTTAC-3′ (reverse)

BS7
Outer: 5′-GATTATTGGTTATATATTTGATTTG-3′ (forward), 5′-AACAACCTCCAAATTATACC-3′ (reverse)
Nested: 5′-TAGAGATAGTGGATGATGT-3′ (forward), 5′-TCAACACACCATCTACC-3′ (reverse)

Fig. S11. Primers for amplify the indicated regions of the human MMP-2 genomic locus.

Region	probe number	HM450 probe ID	CpG site (GRCh37)	Location
BS3	p1	cg08318842	Chr16:55512806	TSS1500
	p2	cg09530163	Chr16:55512822	TSS1500
	p3	cg12317456	Chr16:55512868	TSS1500
	p4	cg01821058	Chr16:55512870	TSS1500
	p5	cg04862799	Chr16:55513017	TSS200
	p6	cg27642062	Chr16:55513148	1stExon;5'UTR
BS4	p7	cg14400118	Chr16:55513191	1stExon;5'UTR
	p8	cg27279620	Chr16:55513660	Body
BS5	p9	cg00078334	Chr16:55513961	Body
BS6	p10	cg07582157	Chr16:55514269	Body
	p11	cg09350341	Chr16:55514378	Body
	p12	cg02458945	Chr16:55514470	Body

Fig. S12. The IDs and locations of the HM450 probes used for TCGA analysis in Fig. 3b–d.

ChIP-a
5′-AAGGGCCTAGAGCGACAGAT-3′ (forward), 5′-TCTACGTCCACCCTCAGTGC-3′ (reverse)

ChIP-b
5′-CTGCCCTCCCTTGTTTCC-3′ (forward), 5′-ATGTTTAAAGCCCCAGATGC-3′ (reverse)

ChIP-c
5′-GCAACCTTCAGCATACAGCA-3′ (forward), 5′-AGAGGATGTCCCCGCTATTT-3′ (reverse)

Fig. S13. Primers for ChIP.

H28 cells

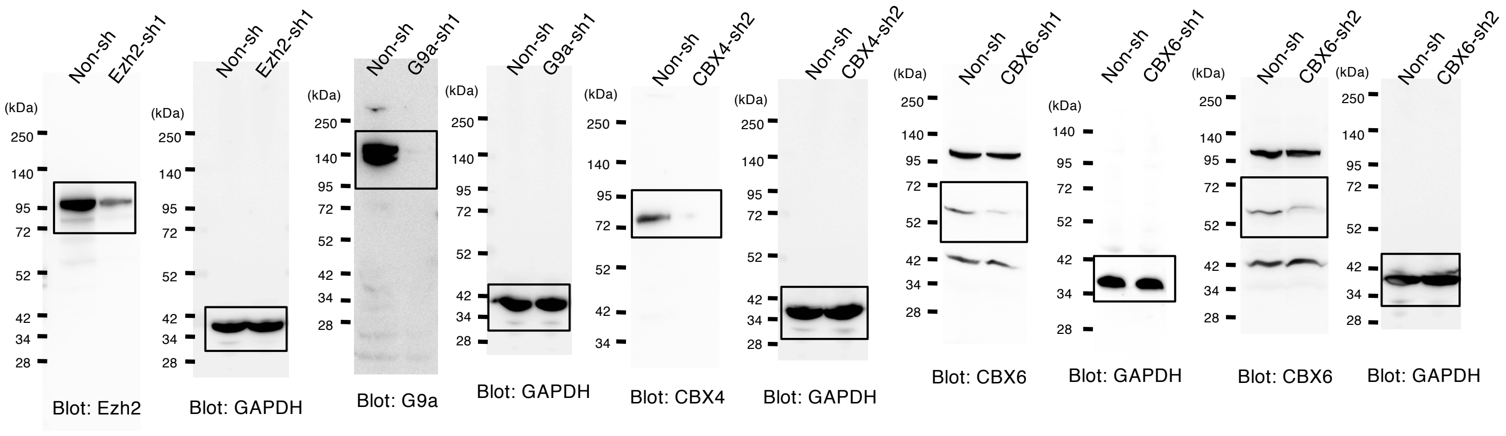

Meso-4 cells

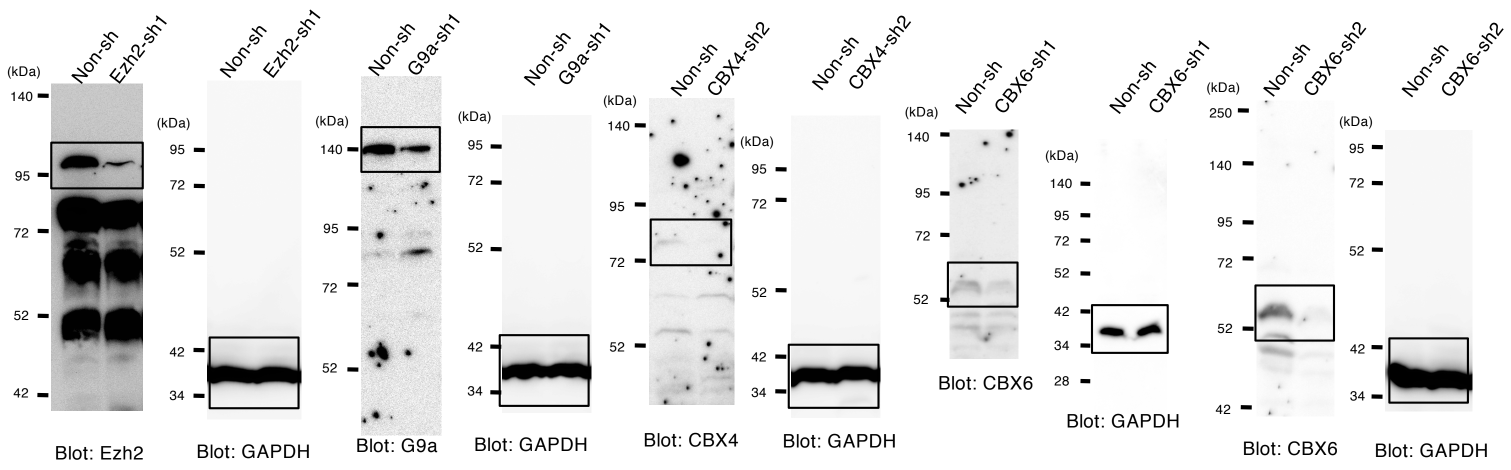

H2052 cells

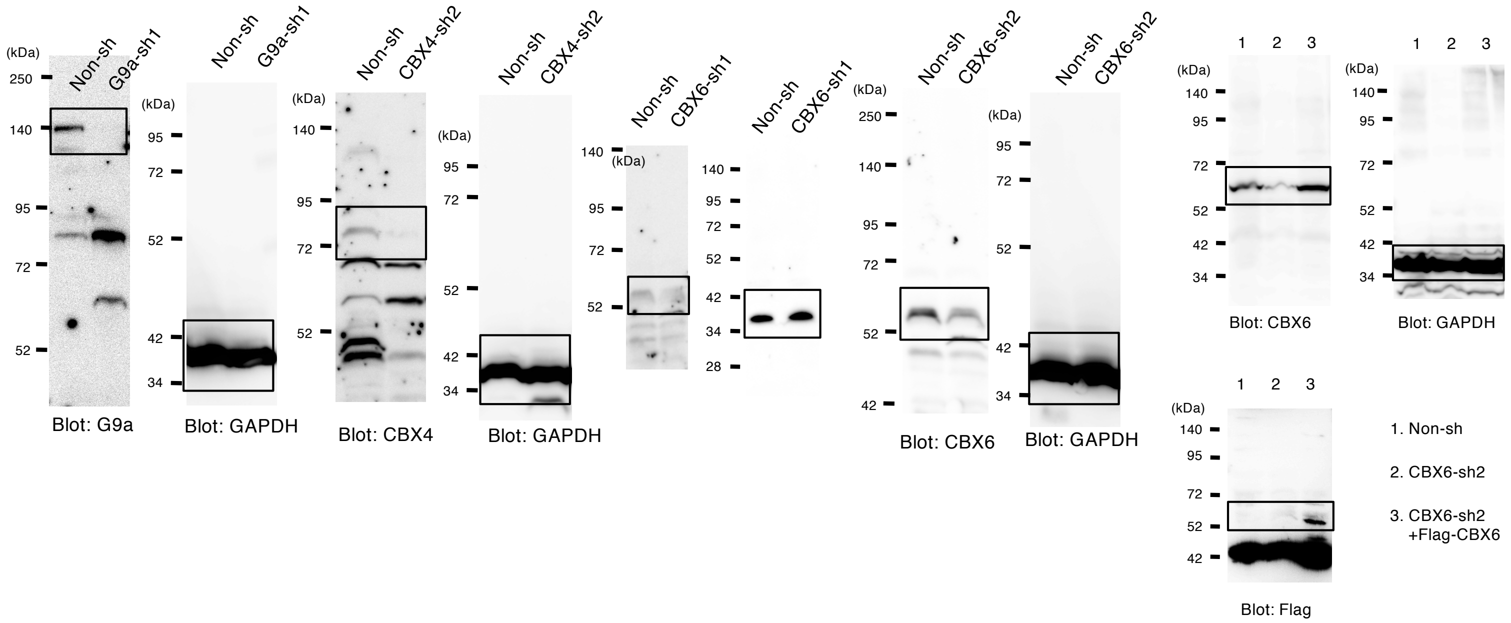

Fig. S14. Full membrane images of Fig. 4b and Fig. 5a.

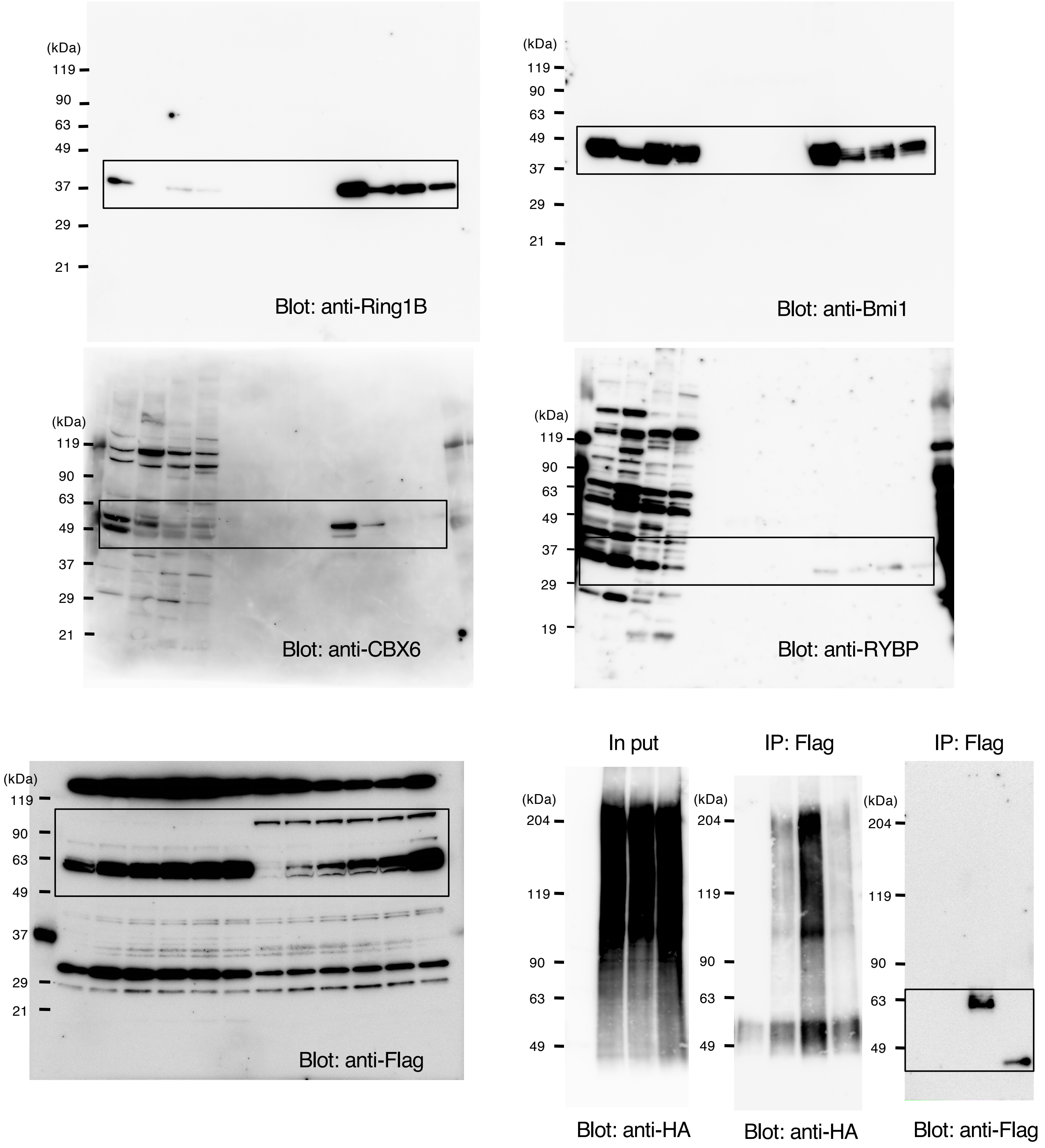

Fig. S15. Full membrane images of Fig. 7a, c, and Fig. 8b.
